# A systematic review and integrated analysis of biologics that target Type 2 inflammation to treat COPD with increased peripheral blood eosinophils

**DOI:** 10.1016/j.heliyon.2022.e09736

**Published:** 2022-06-16

**Authors:** Hiroshi Ohnishi, Masamitsu Eitoku, Akihito Yokoyama

**Affiliations:** a Department of Respiratory Medicine and Allergology, Oko-cho, Kohasu, Nankoku, Kochi, 780-8505, Japan; b Department of Environmental Medicine, Kochi Medical School, Kochi University, Oko-cho, Kohasu, Nankoku, Kochi, 780-8505, Japan

**Keywords:** Benralizumab, Chronic obstructive pulmonary disease, Eosinophil, Exacerbation, Mepolizumab

## Abstract

**Background and aims:**

Biologics that target Type 2 inflammation are effective in reducing exacerbations of severe asthma. We conducted a systematic review and integrated analysis of the efficacy and safety of these biologics in chronic obstructive pulmonary disease (COPD) patients with increased peripheral blood eosinophils.

**Methods:**

Clinical trials of biologics that target Type 2 inflammation in COPD were found using PubMed, the Cochrane Library, and ClinicalTrials.gov. We analyzed the clinical efficacy of anti-IL-5-targeted therapy at approved (benralizumab 30 mg, mepolizumab 100 mg, for severe asthma) and high (benralizumab 100 mg, mepolizumab 300 mg) doses.

**Results:**

Approved benralizumab and mepolizumab doses tended to reduce moderate-to-severe exacerbations by 9% [risk ratio (RR) 0.91, 95% confidence interval (CI) [0.83, 1.00], *p* = 0.05], but did not reduce exacerbations requiring emergency department visits or hospitalization. High-dose benralizumab and mepolizumab reduced moderate-to-severe exacerbations by 12% (RR = 0.88, 95% CI [0.80, 0.98], *p* = 0.02) and exacerbations requiring emergency department visits or hospitalization by 33% (RR = 0.67, 95% CI [0.53, 0.84], *p* = 0.0005). Neither dose improved St. George's Respiratory Questionnaire or COPD Assessment Test scores. The safety of benralizumab and mepolizumab was comparable to placebo.

**Conclusions:**

Benralizumab and mepolizumab have limited efficacy in reducing moderate-to-severe exacerbations in COPD patients with increased peripheral blood eosinophils and requires at least high doses.

## Highlights

• This integrated analysis shows limited efficacy of anti-IL-5 therapy in COPD.
• High doses of anti-IL-5 therapy are required to reduce moderate-to-severe exacerbations.

## 1 Introduction

Chronic obstructive pulmonary disease (COPD) is a common, preventable, and treatable disease characterized by persistent respiratory symptoms and airflow limitation. COPD is caused by airway and/or alveolar abnormalities following exposure to noxious particles such as tobacco smoke [1]. COPD exacerbation is defined as acute worsening of respiratory symptoms such as dyspnea, cough, and/or sputum that requires additional treatment including antibiotics, bronchodilator, and/or systemic corticosteroids; it accounts for a large proportion of the total COPD burden on healthcare costs [1]. A history of two or more exacerbations within the previous year is associated with deteriorating airflow limitation, the future development of exacerbations, hospitalization, poor prognosis, and an increased risk of death [1]. Therefore, a reduction in exacerbations was set as a primary outcome of clinical trials for the efficacy of anti-inflammatory drugs in COPD patients.

Type 2 airway inflammation induced by antigen-specific immunoglobulin E (IgE), interleukin-4 (IL-4), IL-5, IL-13, and thymic stromal lymphopoietin is associated with increased exacerbations and airway remodeling in patients with asthma. Omalizumab, dupilumab, benralizumab, and mepolizumab are monoclonal antibodies (mAbs) against IgE, IL-4/IL-13 signaling, the anti-IL-5 receptor α, and anti-IL-5, respectively. In phase III clinical trials, these four mAbs reduced moderate-to-severe exacerbations in severe asthma patients with Type 2 airway inflammation [2, 3, 4, 5, 6] and are approved to treat severe asthma in Europe, the United States, and Japan. Recently, several biologics were tested in clinical trials for COPD [7, 8, 9, 10, 11], and five systematic reviews and meta-analyses evaluated the efficacy of biologics including benralizumab and mepolizumab on COPD patients [12, 13, 14, 15, 16]. However, these reports had some statistical problems, and/or compared their efficacy in all COPD patients, as detailed in the discussion section. The efficacy of biologics that target Type 2 inflammation should be investigated in COPD patients with Type 2 inflammation. Therefore, we conducted a systematic review and integrated analysis of the efficacy and safety of these biologics in COPD patients with increased peripheral blood eosinophils.

## 2 Methods

### 2.1 Literature search

This systematic review and integrated analysis was performed in accordance with the Preferred Reporting Items for Systematic Review and Meta-Analyses (PRISMA) Statement [17]. We searched for clinical trials of mAbs that target Type 2 inflammation, including omalizumab, mepolizumab, benralizumab, reslizumab, dupilumab, lebrikizumab, tralokinumab, and tezepelumab using the following medical subject heading terms “chronic obstructive pulmonary disease” and/or “COPD” in the PubMed, Cochrane Library, and ClinicalTrials.gov databases on 1 December, 2021.

### 2.2 Inclusion and exclusion criteria

Double-blind, placebo-controlled trials of these mAbs in study subjects with COPD were included. Subjects with asthma COPD overlap (ACO) were excluded. Two reviewers independently validated the relevant studies from the literature searches. The eligible studies were selected by PRISMA criteria and the disagreements regarding eligibility were resolved by consensus. The quality of a body of evidence was assessed using the Grading of Recommendations Assessment, Development, and Evaluation (GRADE) system [18].

### 2.3 Outcome measures

The following outcome measures were included to evaluate clinical efficacy and safety: moderate-to-severe exacerbations, time to the first moderate-to-severe exacerbation, exacerbations leading to an emergency department (ED) visit or hospitalization, the St. George's Respiratory Questionnaire (SGRQ) score, COPD Assessment Test (CAT) score, pre-bronchodilator forced expiratory volume in one second (FEV1), rescue inhaler use, the proportion of nights awoken, all adverse events, serious adverse events, and death. The effect measures used in the outcome synthesis are as follows. The risk ratio (RR) was used for moderate-to-severe exacerbations and exacerbations leading to an ED visit or hospitalization. The hazard ratio (HR) was used for time to first moderate-to-severe exacerbation. The mean difference (MD) was used for the SGRQ score, the CAT score, FEV1, rescue inhaler use, and the proportion of nights awoken. The odds ratio (OR) was used for all adverse events, serious adverse events, and death.

### 2.4 Statistical analysis

The integrated analysis was conducted with the "meta" package of R version 4.1.1. A *p*-value less than 0.05 was considered statistically significant. Statistical heterogeneity among the trials was assessed using the standard *I*^2^ value of 0–25%, 25–50%, 50–75%, and 75–100%, which indicated no, low, moderate, and high heterogeneity, respectively. A fixed-effect model (the inverse variance method for MD and HR as well as the Mantel-Haenszel method for RR and OR) was used when heterogeneity was absent. Otherwise, a random-effect model (the Der Simonian and Laird method) was used. We did not consider publication bias because of the low number of publications included in the study.

## 3 Results

### 3.1 Systematic review of the literature

**Figure 1:**
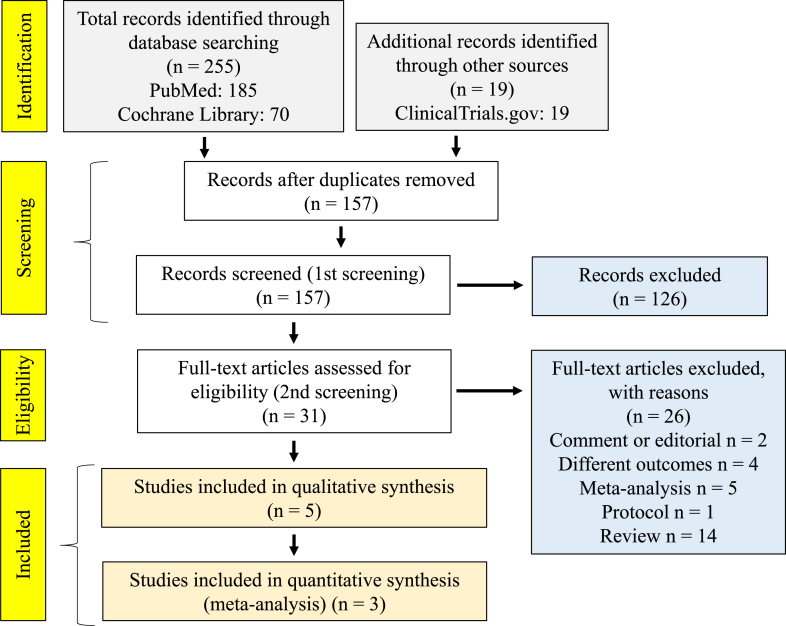
A PRISMA flow diagram for integrated analysis study selection.

A PRISMA flow diagram to select studies for the integrated analysis of biologics that treat COPD with increased peripheral blood eosinophils is shown in Figure 1. One Phase II clinical trial was planned for omalizumab but was withdrawn due to a lack of eligible subjects. No randomized controlled trials (RCTs) of reslizumab or tralokinumab to treat COPD were reported. Lebrikizumab completed a Phase II trial in COPD, but the results were not reported. As of 1 December, 2021, two Phase III RCTs and a Phase II RCT in COPD were planned for dupilumab and tezepelumab, respectively, and patient enrollment was underway. Three RCT results for mepolizumab [(1) NCT01463644 [7], (2) NCT02105948 (METREX), (3) NCT 02105961 (METREO) [8]] and three RCT results for benralizumab in COPD [(1) NCT01227278 [9], (2) NCT02138916 (GALATHEA), (3) NCT02155660 (TERRANOVA) [10, 11]] were reported in five papers.

### 3.2 Basic characteristics of the included studies

To analyze COPD patients with increased peripheral blood eosinophils, all cases reported by Dasgupta, et al. [7] (COPD patients with eosinophils >3% in sputum within the past 2 years), all cases of METREO (COPD patients with peripheral blood eosinophil count ≥150/μL at screening or ≥300/μL in the previous year), a subpopulation of METREX (COPD with peripheral blood eosinophil counts ≥150/μL at screening or ≥300/μL in the previous year) [8], all cases reported by Brightling et al. [9], subpopulations of GALATHEA and TERRANOVA (COPD with peripheral blood eosinophil count ≥220/μL) [10] were included in the integrated analysis. For analyses of serious adverse events, death, and all adverse events, the analysis included all patients with COPD because event data stratified by COPD with increased peripheral blood eosinophils were not available in GALATHEA and TERRANOVA [10, 11]. Baseline characteristics of included studies were listed in Table 1. The study subjects were not only COPD patients with increased peripheral blood eosinophils, but also those with frequent exacerbation that primarily took inhaled corticosteroids, and about 30% of patients who were current smokers.

**Table 1:** Comparison of baseline characteristics for selected COPD patients with increased peripheral blood eosinophils. GOLD = Global Initiative for Chronic Obstructive Lung Disease; Eos = eosinophils; Mepo = mepolizumab; Benra = benralizumab; BD = bronchodilator; ICS = inhaled corticosteroids; LABA = long-acting beta 2 agonist; LAMA = long-acting muscarinic antagonist. References: 1. Pavord ID, et al. *N Engl J Med*. 2017; 377:1613–1629. 2. Brightling CE, et al. *Lancet Respir Med*. 2014; 2:891–901. 3. Criner GJ, et al. *N Engl J Med*. 2019; 381:1023–1034.

Study	METREX1		METREO1			Brightling et al.2		GALATHEA3			TERRANOVA3			
Inclusion criteria for eosinophils	Peripheral blood Eos ≥ 150/μL at screening or ≥300/μL during the previous year					Sputum Eos ≥3%		Peripheral blood Eos ≥ 220/μL at baseline						
	Mepo 100 mg	Placebo	Mepo 100 mg	Mepo 300 mg	Placebo	Benra 100 mg	Placebo	Benra 30 mg	Benra 100 mg	Placebo	Benra 10 mg	Benra 30 mg	Benra 100 mg	Placebo
n	233	229	223	225	226	51	50	382	379	359	377	394	386	388
Age	65 ± 8	65 ± 9	65 ± 9	65 ± 9	66 ± 9	63 ± 8	65 ± 8	66 ± 8	66 ± 8	66 ± 8	65 ± 8	66 ± 8	65 ± 8	65 ± 8
Female (%)	36	34	41	30	31	31	42	29	31	28	33	32	35	35
Current smoker (%)	27	31	25	32	28	33	42	37	34	32	29	27	28	30
Ex-smoker (%)	70	64	73	66	71	67	58	63	66	68	71	73	72	70
GOLD group D (%)	94	95	95	97	96	–	–	–	–	–	–	–	–	–
Exacerbations in the previous year	2.6 ± 1.3	2.5 ± 1.2	2.5 ± 1.2	2.7 ± 1.4	2.7 ± 1.5	1.6 ± 1.0	1.6 ± 1.0	2.3 ± 1.2	2.3 ± 1.2	2.4 ± 1.4	2.3 ± 1.0	2.2 ± 1.0	2.3 ± 1.0	2.3 ± 1.0
Post-BD % predicted FEV1	45 ± 15	43 ± 15	47 ± 15	45 ± 16	46 ± 15	44 ± 16	50 ± 18	42 ± 11	44 ± 12	43 ± 13	44 ± 12	43 ± 12	43 ± 12	43 ± 11
Geometric mean Eos (/mm^3^)	260	290	300	310	310	–	–	–	–	–	–	–	–	–
Baseline mean Eos (/mm^3^)	–	–	–	–	–	249 ± 193	230 ± 165	451 ± 281	459 ± 277	450 ± 283	518 ± 420	503 ± 389	504 ± 404	493 ± 360
ICS/LABA/LAMA (%)	100					50	41	72.3	69.1	67.4	57.3	56.9	61.4	59.0
ICS/LABA (%)	–					10	10	18.8	21.1	24.0	34.7	32.3	34.5	34.3
LABA/LAMA (%)	–					–		8.6	9.8	8.6	7.7	10.7	4.1	6.4
Current asthma (%)	excluded					excluded		4.5	6.9	5.0	1.6	4.3	3.1	4.1
Previous history of asthma (%)	excluded for never smokers with a history of asthma					–		6.8	10.0	8.1	4.8	5.3	7.3	7.2

Finally, the clinical efficacy of anti-IL-5-targeted therapy at approved doses for severe asthma (benralizumab 30 mg, mepolizumab 100 mg) and high (benralizumab 100 mg, mepolizumab 300 mg) doses were included in the integrated analysis of COPD patients with increased peripheral blood eosinophils. In the TERRANOVA, benralizumab 10 mg was administered subcutaneously, but these subjects were not included in the integrated analysis because it was not shown to be effective, it was a lower dose than approved, and was subject to being double counted in the placebo. The pilot study [7] was not included in the final integrated analysis because mepolizumab was administered intravenously at a higher dose of 750 mg, which is not an approved route of administration and the number of subjects was extremely small.

### 3.3 Clinical efficacy of anti-IL-5-targeted therapy: primary outcomes

**Figure 2:**
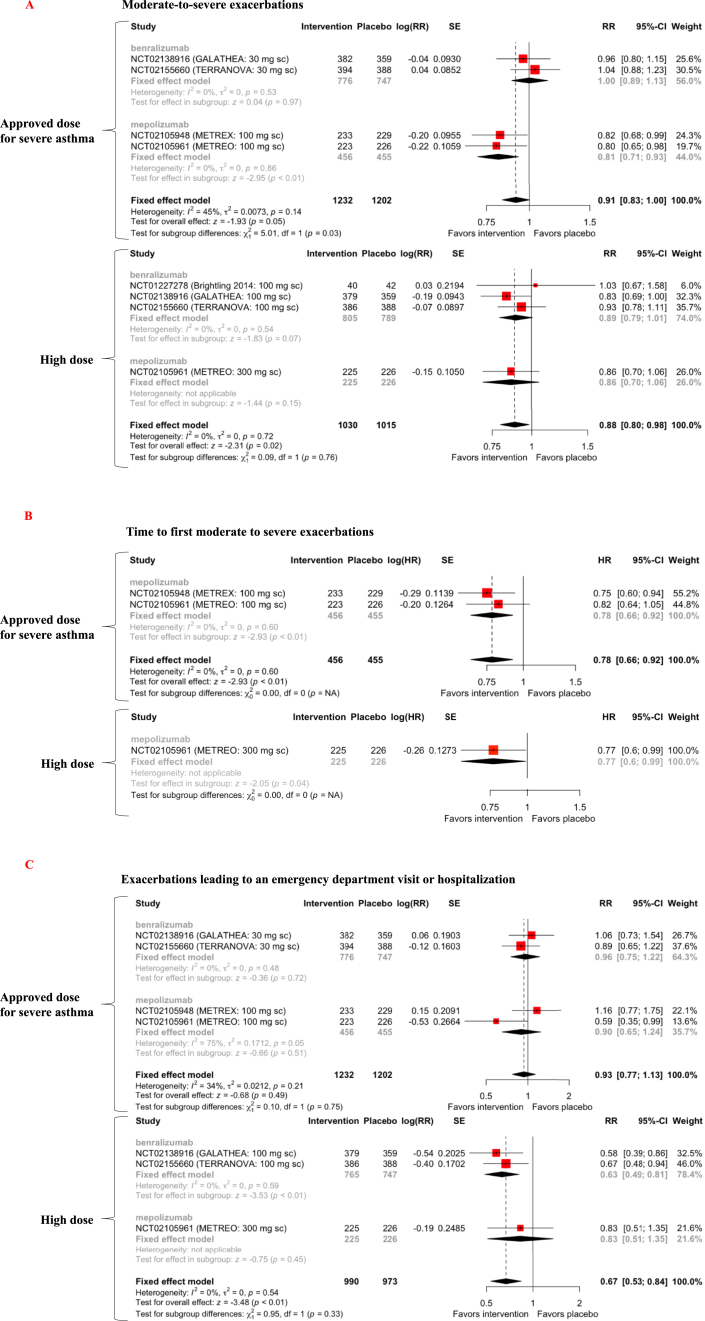
Forest plot of anti-IL-5-targeting therapy effects on moderate-to-severe exacerbations (A), the time to first moderate-to-severe exacerbation (B), and exacerbations leading to an emergency department visit or hospitalization (C) in COPD patients with increased peripheral blood eosinophils. SE = standard error; RR = risk ratio; CI = confidence interval.

In three RCTs of benralizumab for COPD patients (n = 4,012), 2,230 patients received benralizumab, but only 1,581 were COPD patients with increased peripheral blood eosinophils [9, 10, 11]. In three RCTs of mepolizumab for COPD patients (n = 1,530), 865 patients received mepolizumab, but only 681 COPD patients with increased peripheral blood eosinophils received mepolizumab [7, 8]. At the approved dose for severe asthma, benralizumab did not reduce moderate-to-severe exacerbations, whereas mepolizumab reduced moderate-to-severe exacerbations by 19% [RR = 0.81, 95% confidence interval (CI) [0.71, 0.93], *p* = 0.003]. An integrated analysis of approved benralizumab and mepolizumab doses showed a trend of 9% reduction in moderate-to-severe exacerbations (RR = 0.91, 95% CI [0.83, 1.00], *p* = 0.05, level of evidence C) (Figure 2A). At high doses, neither benralizumab nor mepolizumab reduced moderate-to-severe exacerbations, however, an integrated analysis showed a 12% reduction in moderate-to-severe exacerbations (RR = 0.88, 95% CI [0.80, 0.98], *p* = 0.02, level of evidence B) (Figure 2A). Approved and high doses of mepolizumab increased the time to first moderate-to-severe exacerbation by 22% (HR = 0.78, 95% CI [0.66, 0.92], *p* = 0.003, level of evidence B) and 23% (HR = 0.77, 95% CI [0.60, 0.99], *p* = 0.04, level of evidence C), respectively (Figure 2B). An integrated analysis of approved benralizumab and mepolizumab doses did not show a reduction in exacerbations leading to ED visits or hospitalization (level of evidence C) (Figure 2C). High doses of benralizumab reduced exacerbations leading to an ED visit or hospitalization by 37% (RR = 0.63, 95% CI [0.49, 0.81], *p* = 0.0004) but high doses of mepolizumab did not. An integrated analysis of high benralizumab and mepolizumab doses showed a 33% reduction in exacerbations leading to an ED visit or hospitalization (RR = 0.67, 95% CI [0.53, 0.84], *p* = 0.0005, level of evidence B) (Figure 2C).

### 3.4 Clinical efficacy of anti-IL-5-targeted therapy: secondary outcomes

**Figure 3:**
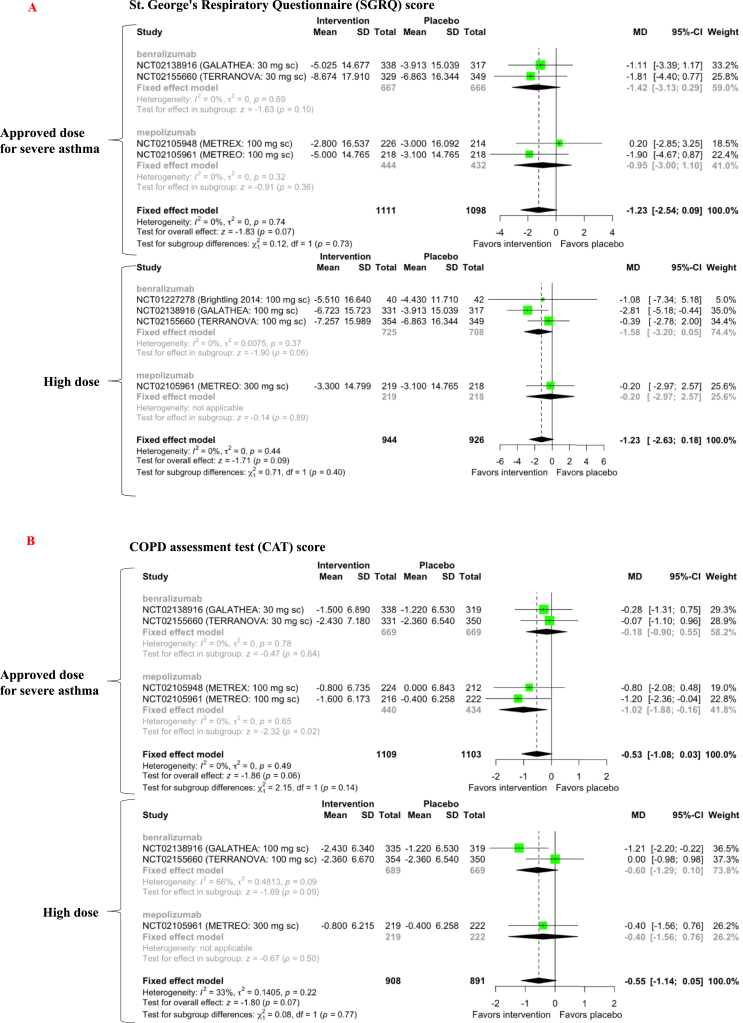
Forest plot of anti-IL-5-targeting therapy effects on St. George's Respiratory Questionnaire (SGRQ) score (A) and the COPD assessment test (CAT) score (B) in COPD patients with increased peripheral blood eosinophils. SD = standard deviation; MD = mean difference; CI = confidence interval.

**Figure 4:**
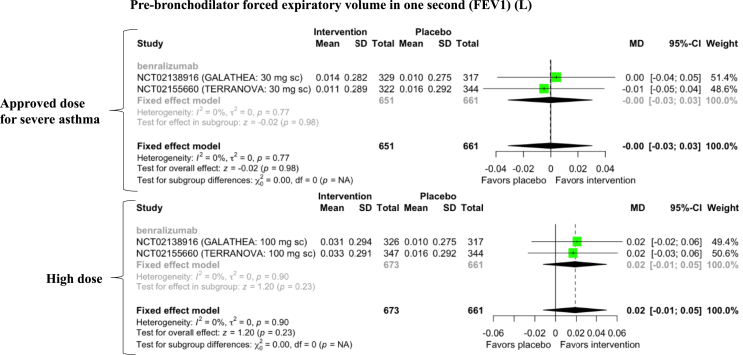
Forest plot of anti-IL-5-targeting therapy effects on pre-bronchodilator forced expiratory volume in one second (FEV1) in COPD patients with increased peripheral blood eosinophils. SD = standard deviation; MD = mean difference; CI = confidence interval.

An integrated analysis of either approved or high doses of benralizumab and mepolizumab did not improve SGRQ scores (level of evidence C) (Figure 3A). The approved dose of mepolizumab improved the CAT score by one point (mean difference (MD) = −1.02, 95% CI [−1.88, −0.16], *p* = 0.02), which was less than the minimal clinically important difference (MCID) of two points [19]. An integrated analysis of approved or high benralizumab and mepolizumab doses showed no improvement in CAT scores (level of evidence C) (Figure 3B). Neither approved nor high benralizumab doses improved pre-bronchodilator FEV1 (level of evidence C) (Figure 4).

**Figure 5:**
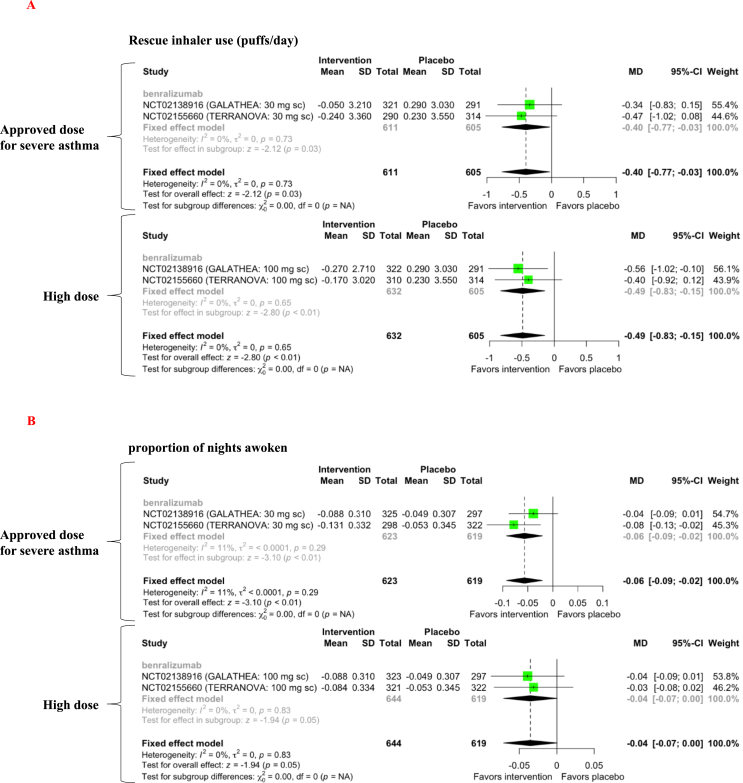
Forest plot of anti-IL-5-targeting therapy effects on rescue inhaler use (A) and the proportion of nights awoken (B) in COPD patients with increased peripheral blood eosinophils. SD = standard deviation; MD = mean difference; CI = confidence interval.

Approved and high benralizumab doses reduced rescue inhaler use by 0.4 puffs/day (MD = −0.40, 95% CI [−0.77, −0.03], *p* = 0.03, level of evidence B) and 0.49 puffs/day (MD = −0.49, 95% CI [−0.83, −0.15], *p* = 0.005, level of evidence B), respectively (Figure 5A). However, these differences were below the MCID. A study evaluating the MCID of rescue inhaler use in COPD patients reported that an MCID of a four-point improvement in the SGRQ score corresponded with a decrease of 0.6 puffs/day and an MCID of a 100-mL improvement in FEV1 was equivalent to a decrease of 1.3 puffs/day [20]. The approved dose of benralizumab reduced the proportion of nights awoken by 6% (MD = −0.06, 95% CI [−0.09, −0.02], *p* = 0.002, level of evidence B). There was a trend for high-dose benralizumab to reduce the proportion of nights awoken (MD = −0.03, 95% CI [−0.07, 0.00], *p* = 0.06, level of evidence C) (Figure 5B).

### 3.5 Clinical safety of anti-IL-5-targeted therapy

**Figure 6:**
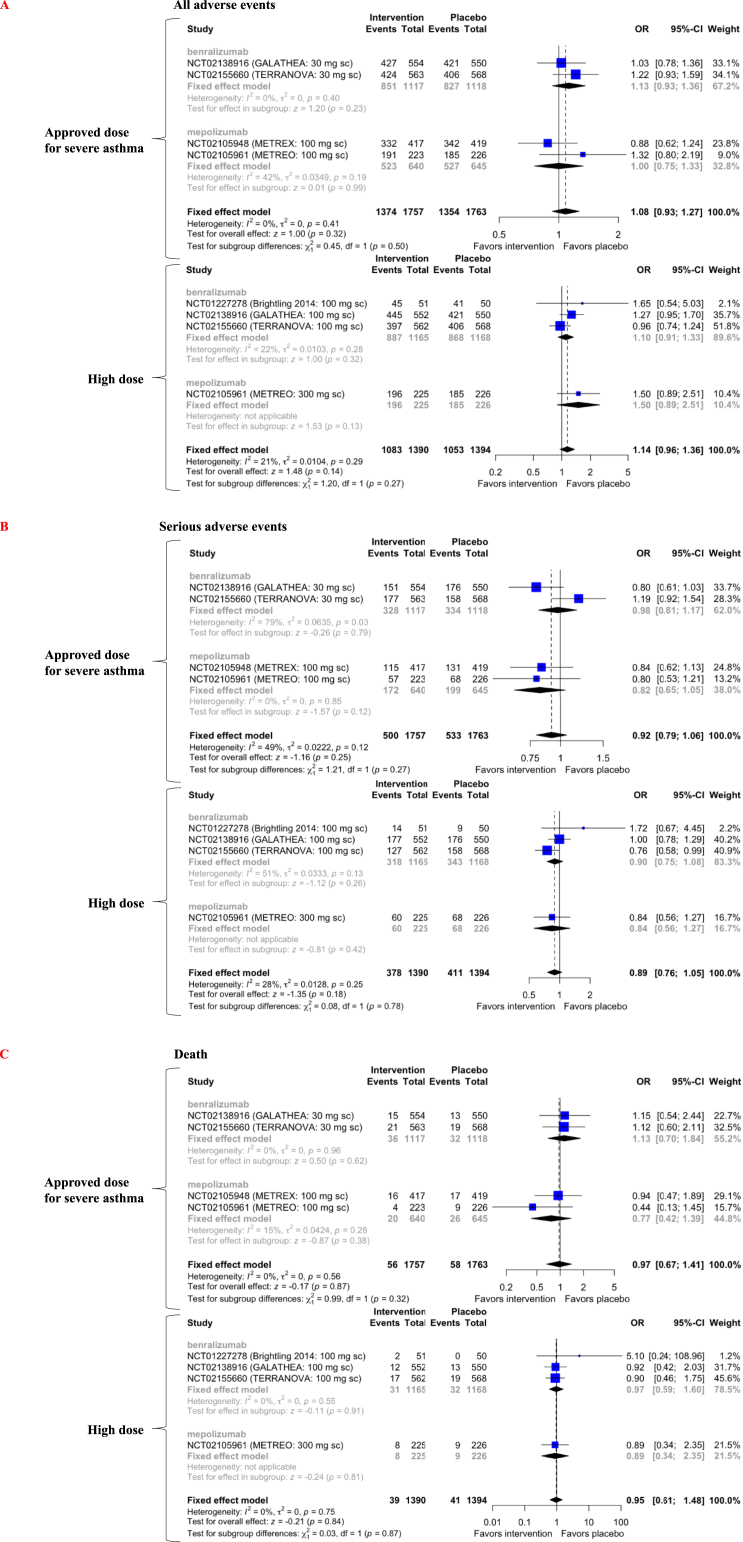
Forest plot of anti-IL-5-targeting therapy effects on all adverse events (A), serious adverse events (B), and death (C) in patients with COPD. OR = odds ratio; CI = confidence interval.

In both approved and high doses of benralizumab and mepolizumab, the incidence of all adverse events, serious adverse events, and death was comparable to placebo in patients with COPD (level of evidence C for serious adverse events, level of evidence B for all adverse events and death) (Figure 6A–C).

## 4 Discussion

In the present systematic review and integrated analysis, we showed statistically significant but limited benralizumab and mepolizumab efficacy at reducing moderate-to-severe exacerbations in COPD patients with increased peripheral blood eosinophils.

Approved doses of benralizumab and mepolizumab tended to reduce moderate-to-severe exacerbations by 9% but did not reduce exacerbations requiring ED visits or hospitalization or improve SGRQ or CAT scores. High doses of benralizumab and mepolizumab reduced moderate-to-severe exacerbations by 12% and exacerbations leading to an ED visit or hospitalization by 33% but did not improve SGRQ or CAT scores. These data clearly demonstrate that high doses are required for anti-IL-5-targeting therapy to alleviate moderate-to-severe exacerbations in COPD patients with increased peripheral blood eosinophils. When discussing clinical efficacy, it should be noted that study subjects were not only COPD patients with increased peripheral blood eosinophils, but also those with frequent exacerbation mostly taking inhaled corticosteroids and about 30% of patients were current smokers (Table 1).

Five meta-analyses for biologics in patients with COPD were published to-date. Rogliani et al. conducted a meta-analysis of biologics with differing mechanisms of action, including anti-IL-5, anti-IL-5 receptor α, ant-IL-1β, anti-IL-1 receptor 1, anti-IL-8, and anti-tumor necrosis factor-α mAbs [12]. Unfortunately, their analysis double counted placebos in the three-group comparison of the METREO trial for mepolizumab. Lan, et al. resolved this double-counting problem by comparing the placebo with an integration of approved and high doses. However, their analysis included all COPD patients, making the effect of benralizumab and mepolizumab on COPD with increased peripheral blood eosinophils less detectable [13]. Donovan et al. conducted a dose-specific analysis of mepolizumab and benralizumab. However, there is no integrated analysis of both mepolizumab and benralizumab and there are inconsistencies with the detailed data reported on ClinicalTrial.gov, which raises questions about the accuracy of the analyses [14]. Pavord et al. reported a pre-specified meta-analysis of mepolizumab in COPD patients with increased peripheral blood eosinophils in the METREX and METREO studies and showed that the approved dose of mepolizumab reduced moderate-to-severe exacerbation by 18%, but the analysis did not consider benralizumab [15]. Zhang, et al. reported an integrated analysis of mepolizumab and benralizumab in eosinophilic COPD, which also double counted placebo in the three-group comparison in the METREO trial for mepolizumab [16]. Some previous reports considered the study subjects to have eosinophilic COPD [8, 16], but this tentative name is confused with COPD with eosinophilic airway inflammation or ACO.

The major differences between previous meta-analyses and the present study are described below. First, we conducted an integrated analysis of approved doses, those approved for severe asthma, and high doses in COPD patients with increased peripheral blood eosinophils. High doses of benralizumab and mepolizumab did not prevent the primary endpoint of moderate-to-severe exacerbations, in their respective clinical trials, but when the two drugs were integrated for the analysis, moderate-to-severe exacerbations decreased by 12%. Second, the data for benralizumab, which was not included in the original paper, was included as a secondary endpoint in the integrated analysis for exacerbations leading to an ED visit or hospitalization. By combining the detailed data reported in the CrinicalTrials.gov, the high-dose benralizumab or mepolizumab group had a 33% reduction in exacerbations leading to an ED visit or hospitalization. Third, previous meta-analyses concluded that anti-IL-5-targeted therapy significantly reduced moderate-to-severe exacerbations in COPD, but our integrated analysis of two doses demonstrated that the doses approved for severe asthma were not sufficient. In our analysis, high doses, at a minimum, were required to reduce moderate-to-severe exacerbations in COPD patients with increased peripheral blood eosinophils. Finally, drug costs for these biologics are extremely expensive. Approved doses of 100 mg mepolizumab costs about $1,550 every 4 weeks, and 30 mg benralizumab costs about $3,100 three times a month and $1,550 every 8 weeks. Therefore, the clinical benefits of biologics do not appear to be worth their cost for COPD patients with increased peripheral blood eosinophils.

There are several limitations to our study. First, multiple cutoff values for peripheral blood eosinophils were used to define COPD patients with increased peripheral blood eosinophils, as mentioned in the results. Second, we did not evaluate publication bias with a funnel plot analysis because of the few studies used for the integrated analysis.

## 5 Conclusions

The anti-IL-5-targeting biologics benralizumab and mepolizumab have limited efficacy in reducing moderate-to-severe exacerbations in COPD patients with increased peripheral blood eosinophils. Given their high costs, we do not recommend prescribing benralizumab or mepolizumab for COPD patients with increased peripheral blood eosinophils that do not have asthma complications.

## Declarations

### Author contribution statement

Hiroshi Ohnishi: Conceived and designed the experiments; Analyzed and interpreted the data; Wrote the paper.

Masamitsu Eitoku: Analyzed and interpreted the data; Wrote the paper.

Akihito Yokoyama: Conceived and designed the experiments; Wrote the paper.

### Declaration of interest’s statement

The authors declare the following conflict of interests: Hiroshi Ohnishi and Akihito Yokoyama receive lecture fees from pharmaceutical companies including AstraZeneca K.K., Japan, GlaxsoSmithKline plc, Japan, Novartis Pharma, Japan, and Sanofi K.K., Japan, which supply biologics for severe asthma.

### Data availability statement

Data included in article/supp. material/referenced in article.

### Funding statement

This research did not receive any specific grant from funding agencies in the public, commercial, or not-for-profit sectors.

### Additional information

No additional information is available for this paper.
